# CD69 and SBK1 as potential predictors of responses to PD-1/PD-L1 blockade cancer immunotherapy in lung cancer and melanoma

**DOI:** 10.3389/fimmu.2022.952059

**Published:** 2022-08-15

**Authors:** Zhang-Wei Hu, Wei Sun, Yi-Hui Wen, Ren-Qiang Ma, Lin Chen, Wen-Qing Chen, Wen-Bin Lei, Wei-Ping Wen

**Affiliations:** ^1^ Department of Otolaryngology, The First Affiliated Hospital, Sun Yat-sen University, Guangzhou, China; ^2^ Otorhinolaryngology Institute, Sun Yat-sen University, Guangzhou, China; ^3^ Department of Otolaryngology, the Sixth Affiliated Hospital of Sun Yat-sen University, Guangzhou, China

**Keywords:** CD69, SBK1, tumor microenvironment, PD-1, immunotherapy

## Abstract

**Background:**

PD-1/PD-L1 blockade is a promising immunotherapeutic strategy with the potential to improve the outcomes of various cancers. However, there is a critically unmet need for effective biomarkers of response to PD-1/PD-L1 blockade.

**Materials and methods:**

Potential biomarkers of response to PD-1/PD-L1 blockade were obtained from the Cancer Treatment Response gene signature Database (CTR-DB). A comprehensive pan-cancer analysis was done on The Cancer Genome Atlas (TCGA) and Genotype-Tissue Expression (GTEx) datasets. Correlations between gene expression and infiltration by immune cells were assessed using TIMER, EPIC, MCPcounter, xCell, CIBERSORT, and quanTIseq. Immunophenoscore (IPS) was used to assess the potential application of the biomarkers to all TCGA tumors.

**Results:**

Analysis of CTR-DB data identified *CD69* and *SBK1* as potential biomarkers of response to PD-1/PD-L1 blockade. Correlation analysis revealed that in various TCGA cancer datasets, *CD69* expression level correlated positively with most immune checkpoints and tumor-infiltrating immune cells, while *SBK1* expression level correlated negatively with infiltrating immune cells. IPS analysis demonstrated the ability of *CD69* and *SBK1* to predict PD-1/PD-L1 blockade responses in various cancers.

**Conclusion:**

*CD69* and *SBK1* are potential predictors of response to cancer immunotherapy using PD-1/PD-L1 blockade. These biomarkers may guide treatment decisions, leading to precise treatment and minimizing the waste of medical resources.

## Introduction

The development of immune checkpoint blockade therapy, especially strategies that target PD-1 or PD-L1, has revolutionized the treatment of various cancers (1). Several antibodies for blocking PD-1 (such as pembrolizumab and nivolumab) and PD-L1 (such as atezolizumab and avelumab) are FDA approved for clinical use against various cancers, including head and neck cancer, melanoma, and lung cancer (2–5). Several clinical trials have associated PD-1/PD-L1 blockade immunotherapy with superior prognosis when compared with standard chemotherapy (6, 7).

However, only 20%–40% of cancer patients achieve sustained response to PD-1/PD-L1 blockade immunotherapy (8–10) and some patients have been found to experience cancer hyper-progression (11). Clinically, tumor proportional score (TPS) and combined positive score (CPS) based on immunohistochemical analysis of PD-L1 levels are the most widely used predictors of response to PD-1/PD-L1 blockade. However, it has limitations (9) and the development of robust biomarkers remains a significant challenge. Thus, effective biomarkers are still urgently needed for guiding treatment decisions.

Here, we mined data on the Cancer Treatment Response gene signature Database (CTR-DB) (12) and identified candidate biomarkers. We then performed a comprehensive pan-cancer analysis through The Cancer Genome Atlas (TCGA) (13) with the aim of uncovering potential biomarkers of clinical response to PD-1/PD-L1 blockade cancer immunotherapy, which may be helpful to improve the prediction accuracy in clinic.

## Materials and methods

### Data sources

Data on gene expression levels and responses to PD-1/PD-L1 blockade immunotherapy were obtained from CTR-DB (12). Three lung cancer datasets (CTR_RNAseq_13, CTR_RNAseq_197, and CTR_RNAseq_381) and 10 melanoma datasets (CTR_RNAseq_11, CTR_RNAseq_96, CTR_RNAseq_165, CTR_RNAseq_178, CTR_RNAseq_179, CTR_RNAseq_189, CTR_RNAseq_225, CTR_RNAseq_289, CTR_RNAseq_370, CTR_RNAseq_502) were downloaded from CTR-DB and analyzed. All patients involved in the datasets had been treated with PD-1/PD-L1 blockade immunotherapy only. Data for pan-cancer analysis were obtained from TCGA *via* UCSC Xena (14). Corresponding normal tissue gene expression data were downloaded from Genotype-Tissue Expression (GTEx) program (15). Because these resources are publicly available, their use did not require approval by the local ethics committee.

### Differentially expressed genes

Differentially expressed genes (DEGs) were identified using negative binomial distribution analysis with adjusted p-value (Benjamini–Hochberg multiple testing correction method) using the R package, DESeq2. Batch effect removal was done by the “ComBat_seq” function of the “SVA” R package. Log2 (fold change) = >1 and q-value = <0.01 were used as cutoff thresholds for identifying DEGs. Gene set enrichment analysis (GSEA) was implemented through Kyoto Encyclopedia of Genes and Genomes (KEGG).

### Protein–protein interaction

Protein–protein interaction (PPI) analysis was done using the GeneMANIA (16), which offers a biological network integration method of predicting gene function. For genes that were differentially expressed in the response versus non-response group, the top 10 upregulated protein-coding genes (based on log FC value) were subjected to PPI analysis. For similar genes identified using GEPIA2 (17), the top 10 protein-coding genes (based on Pearson’s correlation coefficient) were subjected to PPI analysis.

### Prognosis analysis

Correlation between the gene expression levels and overall survival (OS) was determined using the Cox proportional hazards regression model and log-rank test using the R package, SURVIVAL. Tumors with significance (p<0.01) were then subjected to Receiver Operating Characteristic (ROC) curve analysis using the R package, pROC. The candidate biomarkers of response to PD-1/PD-L1 blockade cancer immunotherapy were subjected to Area Under the Curve (AUC) analysis using pROC and statistical differences calculated using one sample t test.

### Immune checkpoint genes and immune score

The correlation between candidate biomarkers and 60 immune checkpoint genes (18) was analyzed using Pearson’s correlation coefficient. Immune score, stromal score and ESTIMATE (19) (Estimation of STromal and Immune cells in MAlignant Tumour tissues using Expression data) score of 9620 samples across 33 TCGA tumors were calculated using the R package, ESTIMATE and their correlation with the expression levels of candidate biomarkers evaluated using Pearson’s correlation coefficient.

### Immune cell infiltration, immunophenoscore and tumor mutation burden

Tumor-infiltrating immune cell levels were analyzed using Tumor Immune Estimation Resource (TIMER) (20), Estimating the Proportion of Immune and Cancer cells (EPIC) (21), Microenvironment Cell Populations-counter (MCPcounter) (22), xCell (23), Cell-type Identification By Estimating Relative Subsets Of RNA Transcripts (CIBERSORT) (24) and QuanTIseq (25). The IPS (26) and tumor mutation burden (TMB) were analyzed using the R package IOBR and MAFTOOLS separately. Correlation analysis was done using Pearson’s correlation coefficient.

## Results

### 283 DEGs in lung cancer and 133 DEGs in melanoma are identified

**Figure 1:**
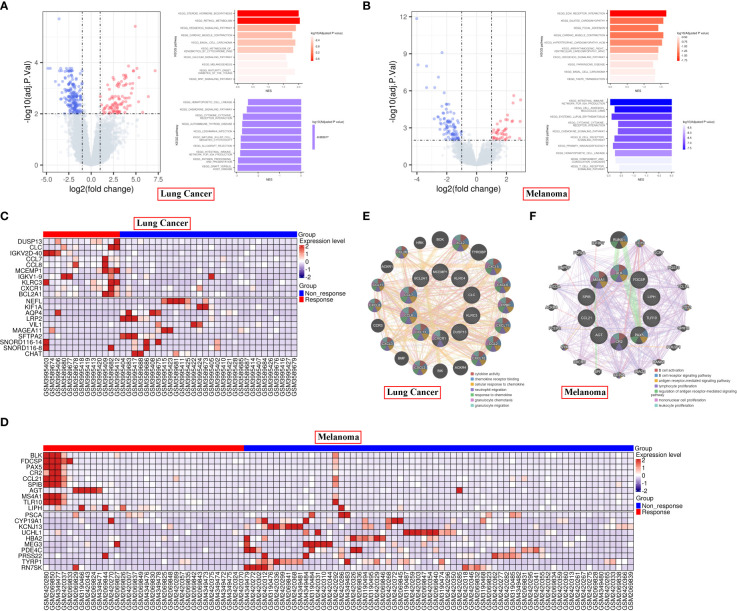
Differential gene expression in responder vs non-responder patients with lung cancer or melanoma. **(A, B)** Volcano plot and KEGG pathways of the DEGs in lung cancer **(A)** and melanoma **(B)**. **(C, D)** Heatmap of the top 10 significantly upregulated or downregulated genes in lung cancer **(C)** and melanoma **(D)**. **(E, F)** PPI analysis of the top 10 upregulated protein-coding genes in lung cancer **(E)** and melanoma **(F)** responders.

First, we analyzed the DEGs between responders and non-responders for lung cancer and melanoma separately. In total, 13 responders and 30 non-responders of lung cancer and 34 responders and 66 non-responders of melanoma were included in the present analysis. In all, we identified 283 DEGs in lung cancer and 133 DEGs in melanoma (**Supplementary Table 1**). GSEA revealed that for lung cancer, the DEGs were mainly enriched for hematopoietic cell lineage and steroid hormone biosynthesis; and that for melanoma, they were enriched for intestinal immune network for IgA production and ECM receptor interaction (**Figures 1A, B**). The top 10 significantly upregulated and downregulated genes are shown in **Figure 1C** for lung cancer and **Figure 1D** for melanoma. GeneMANIA analysis revealed that in lung cancer and melanoma responders, the top 10 upregulated protein-coding genes were primarily associated with cytokine activity and B cell activation, respectively (**Figures 1E, F**).

### *CD69* and *SBK1* are potential predictors of responses to PD-1/PD-L1 blockade

**Figure 2:**
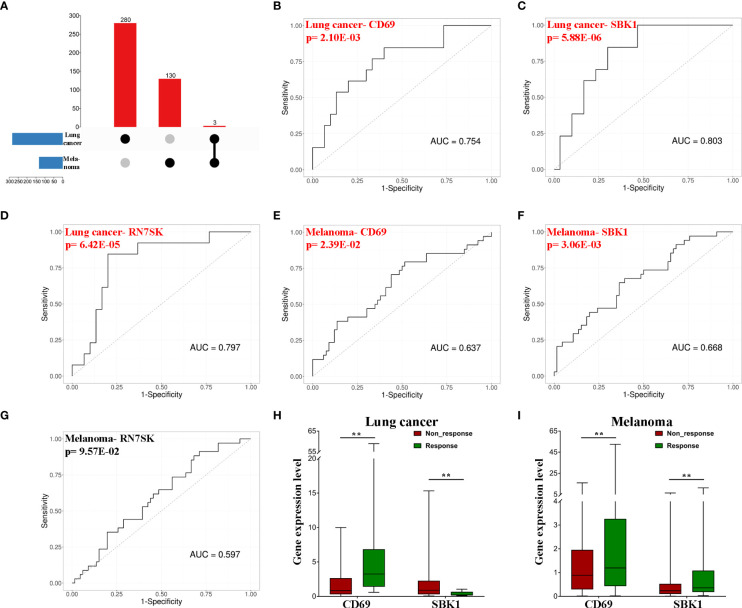
*CD69* and *SBK1* are potential biomarkers of response to PD-1/PD-L1 blockade immunotherapy. **(A)** Up-set of common genes between lung cancer and melanoma responders. **(B–G)** Separate ROC analysis of the 3 common genes in lung cancer and melanoma. **(H)** *CD69* (|LogFC|=2.45, adjusted p-value=4.40E-03) is upregulated in lung cancer responders (responders 8.05 ± 4.33, non-response 1.84 ± 0.44), while *SBK1* (|LogFC|=2.04, adjusted p-value=1.11E-03) is downregulated (responders 0.39 ± 0.09, non-response 1.80 ± 0.53). **(I)** both *CD69* (|LogFC|=1.63, adjusted p-value=3.04E-03, responders 6.34 ± 2.47, non-response 1.76 ± 0.36) and *SBK1* (|LogFC|=1.3, adjusted p-value=7.16E-03, responders 1.26 ± 0.44, non-response 0.51 ± 0.12) are upregulated in melanoma responders. ** indicates p<0.01.

Next, we sought to identify potential biomarkers of pan-cancer response to PD-1/PD-L1 blockade immunotherapy. Based on the intersection, *CD69*, *SBK1* and *RN7SK* were identified as candidate biomarker genes (**Figure 2A**). ROC analysis revealed that AUC values for the ability of *CD69*, *SBK1* and *RN7SK* to predict response to PD-1/PD-L1 blockade immunotherapy in lung cancer were 0.754, 0.803, and 0.797, respectively (**Figures 2B–D**), while in melanoma they were 0.637, 0.668, and 0.597, respectively (**Figures 2E–G**). However, the ability of *RN7SK* to predict melanoma response was not statistically significant (**Figure 2G**, p=0.0957). Thus, *CD69* and *SBK1* were regarded as candidate biomarkers. Further analysis showed that in lung cancer responders, *CD69* was upregulated while *SBK1* was downregulated and that in melanoma responders, both were upregulated (**Figures 2H, I**).

### *CD69* and *SBK1* are aberrantly expressed in most tumors

**Figure 3:**
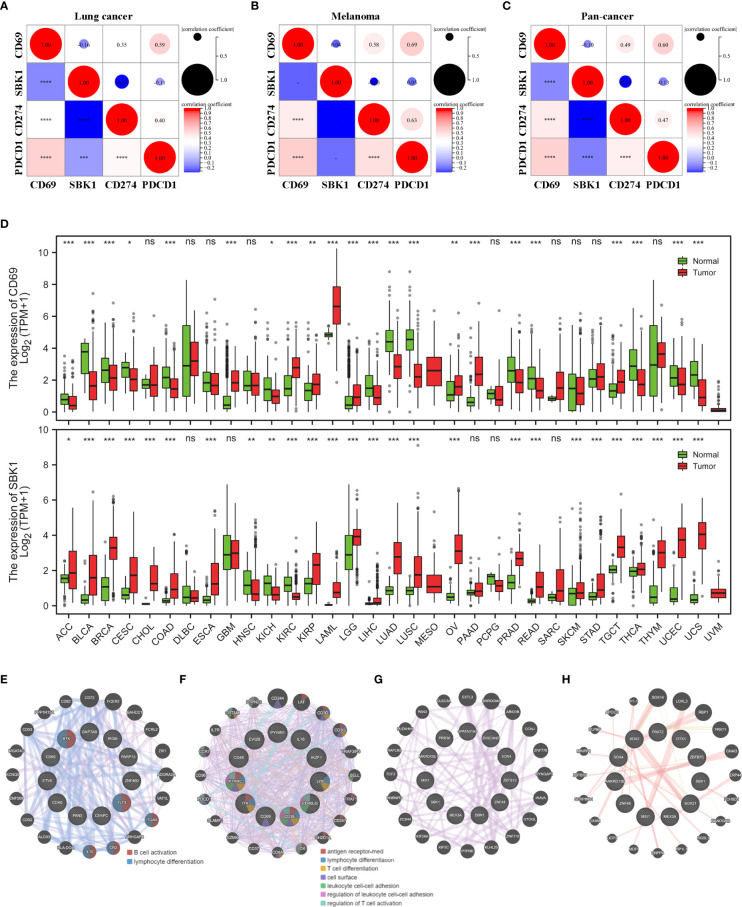
*CD69* and *SBK1* are aberrantly expressed in most TCGA tumors. **(A, C)** Separate analysis of correlation between *CD69*, *SBK1*, *PD-L1* (*CD274*), and *PD-1* (*PDCD1*) expression in TCGA lung cancer **(A)**, melanoma **(B)**, and pan-cancer **(C)**. **(D)** Differences in the expression of *CD69* and *SBK1* in TCGA tumors versus corresponding normal tissues. **(E, F)** PPI analysis of *CD69* similar genes in its 8 upregulated **(E)** and 14 downregulated **(F)** tumors. **(G, H)** PPI analysis of SBK1 similar genes in its 23 upregulated **(G)** and 3 downregulated **(G)** tumors. – indicates not significant (ns). * indicates p<0.05. ** indicates p<0.01. *** indicates p<0.001. **** indicates p<0.0001.

We then compared the mRNA levels of *CD69* and *SBK1* in tumor versus normal tissues using TCGA and GTEx datasets. Analysis of TCGA lung cancer datasets (LUAD and LUSC) showed that while *CD69* exhibited positive correlation with PD-1 and PD-L1, *SBK1* exhibited negative correlation (**Figure 3A**). Similar observations were made upon pan-cancer data analysis (**Figure 3C**). Analysis of melanoma datasets (SKCM and UVM) showed that *CD69* expression positively correlated with PD-1 and PD-L1 but the correlation between *SBK1* and PD-1 or PD-L1 was not significant (**Figure 3B**).

Out of 33 TCGA tumors, *CD69* was upregulated in 8 and downregulated in 14, while *SBK1* was upregulated in 23 and downregulated in 3 (**Figure 3D**). Through GEPIA2 we screened the similar expression genes of *CD69* and *SBK1* (**Supplementary Table 2**). PPI analysis showed that in tumors with upregulated *CD69*, the similar genes were primarily related to B cell activation and lymphocyte differentiation (**Figure 3E**) and that in tumors with downregulated *CD69*, the similar genes were primarily related to antigen receptor-mediated signaling pathway and lymphocyte differentiation (**Figure 3F**). However, no meaningful function entry of *SBK1* similar genes was found in neither *SBK1* upregulated (**Figure 3G**) nor downregulated (**Figure 3H**) tumors.

### *CD69* and *SBK1* are prognostic factors in various TCGA tumors

**Figure 4:**
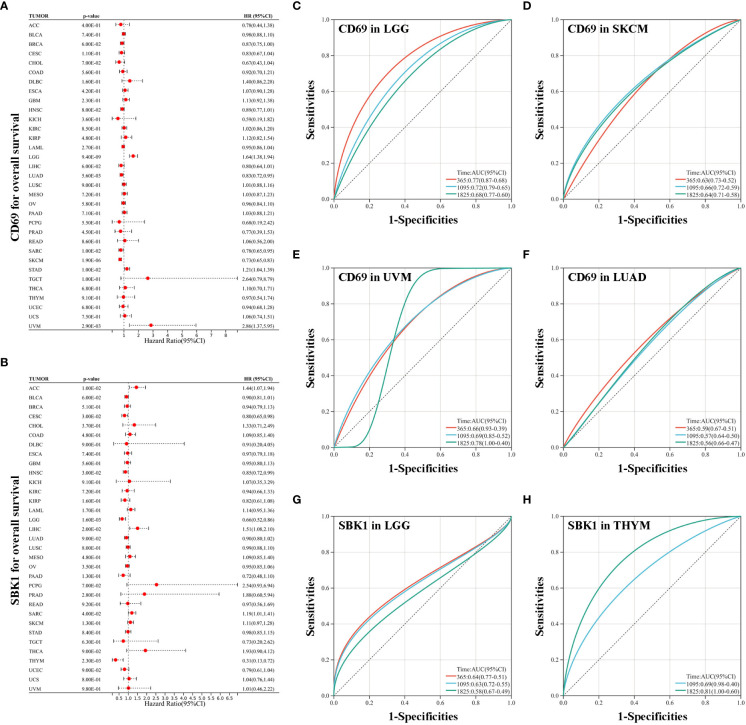
Analysis of the correlation between survival and the expression levels of *CD69* and *SBK1*. **(A)** *CD69* expression levels correlate with poor prognosis in LGG, STAD and UVM, and with improved prognosis in LUAD, SARC and SKCM. **(B)** *SBK1* expression levels correlate with poor prognosis in ACC, LIHC and SARC, and with improved prognosis in CESC, LGG and THYM. **(C–F)** ROC analysis of the ability of *CD69* to predict the 1-, 3-, and 5-year OS of LGG **(C)**, SKCM **(D)**, UVM **(E)**, and LUAD **(F)**. **(G, H)** ROC analysis of the ability of *SBK1* to predict the 1-, 3-, and 5-year OS of LGG **(G)** and THYM **(H)**.

Analysis of the correlation between the mRNA levels of *CD69* and *SBK1*, and overall survival in TCGA tumors revealed that high *CD69* expression correlated with poor prognosis in LGG, STAD, and UVM, and with better prognosis in LUAD, SARC and SKCM (**Figure 4A**). High *SBK1* levels correlated with poorer prognosis in ACC, LIHC and SARC, and with better prognosis in CESC, LGG and THYM (**Figure 4B**). Data on ROC analysis on tumors with p<0.01 following univariate analysis are shown in **Figures 4C–H**.

### *CD69* and *SBK1* levels correlate with the levels of immune checkpoints

**Figure 5:**
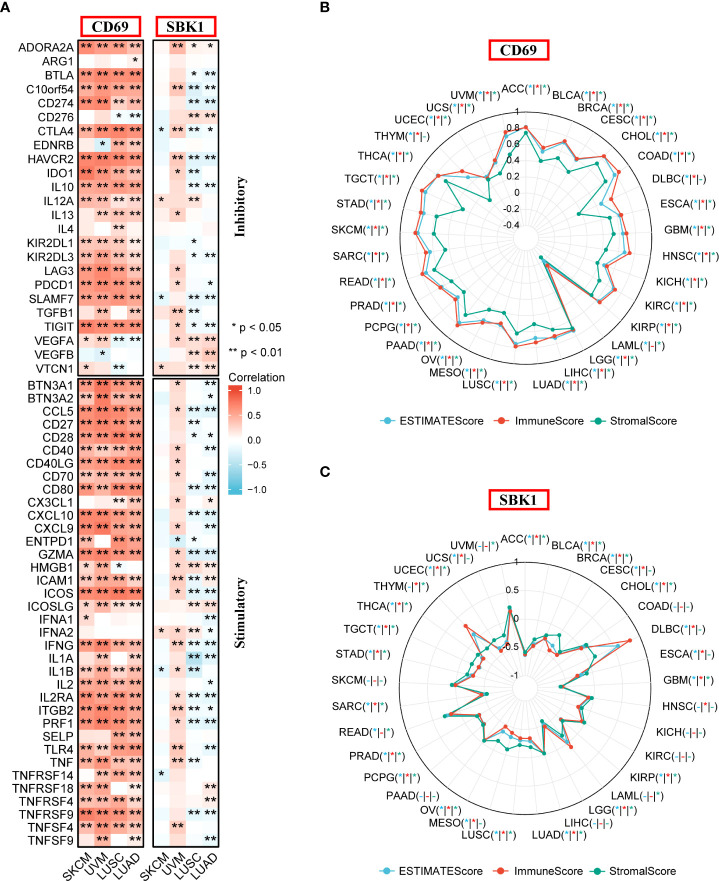
Analysis of the correlation between *CD69* and *SBK1* expression levels and immune checkpoints and ESTIMATE score. **(A)** Correlation between immune checkpoints and the expression levels of *CD69* and *SBK1* in Lung Cancer and Melanoma related TCGA cohorts. **(B, C)** ESTIMATE analysis of *CD69* **(B)** and *SBK1* **(C)** in TCGA tumors. – indicates not significant. * indicates p<0.05. ** indicates p<0.01.

Next, analysis of the correlation between the levels of *CD69* and *SBK1*, and the levels of immune checkpoint genes revealed that *CD69* mRNA levels positively correlated with the levels of most immune checkpoints in most TCGA tumors (**Figure 5A**, **Supplementary Figure 1A**, **Supplementary Table 3**). Interestingly, the levels of *SBK1* mRNA exhibited positive or negative correlation with immune checkpoints in different tumors (**Figure 5A**, **Supplementary Figure 1B**, **Supplementary Table 3**). ESTIMATE analysis showed that the expression levels of *CD69* correlated positively with ESTIMATE score and immune Score in 32 of 33 TCGA tumors (except LAML) (**Figure 5B**), and that *SBK1* levels negatively correlated with ESTIMATE score and immune score in 21 of 33 tumors (**Figure 5C**).

### *CD69* and *SBK1* levels correlate with immune cell levels, IPS and TMB

**Figure 6:**
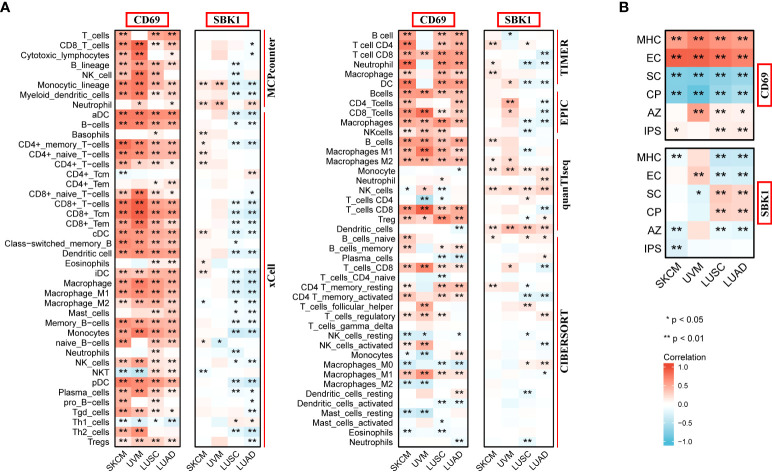
Analysis of correlation between the levels of *CD69* and *SBK1*, and infiltrating immune cells and IPS. **(A)** *CD69* positively correlates with various immune cells in SKCM, UVM, LUSC and LUAD, while *SBK1* negatively correlates with various immune cells in Lung Cancer related cohorts LUSC and LUAD. **(B)** *CD69* levels positively correlate with IPS in SKCM, LUSC and LUAD, while *SBK1* levels negatively correlate with IPS in SKCM. MHC, major histocompatibility complex; CP, immune checkpoints; EC, effector cells; SC, suppressor cells; AZ, average z-scores. * indicates p<0.05. ** indicates p<0.01.

**Figure 7:**
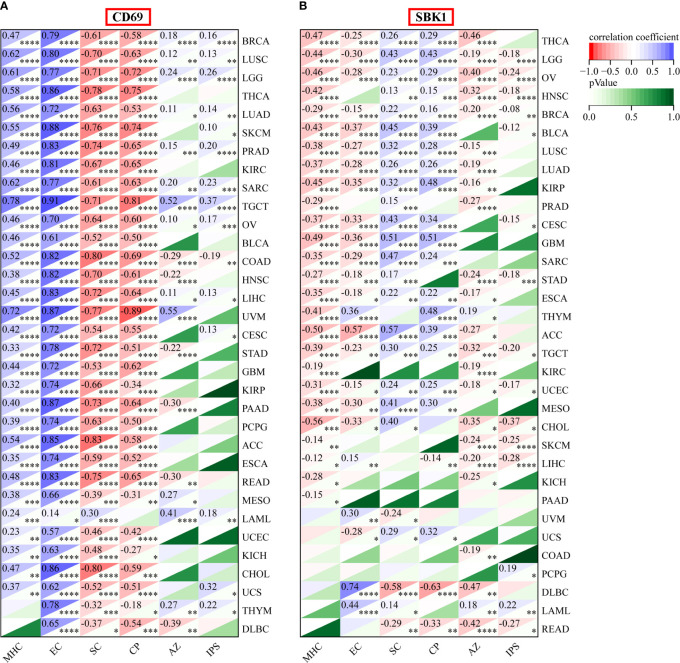
IPS analysis of *CD69* and *SBK1* in TCGA tumors. **(A)** *CD69* levels positively correlate with IPS in various tumors. Top left corner: correlation coefficient. Lower right corner: p-value. **(B)** *SBK1* levels negatively correlate with IPS in various tumors. MHC: major histocompatibility complex, CP, immune checkpoints; EC, effector cells; SC, suppressor cells; AZ, average z-scores. * indicates p<0.05. ** indicates p<0.01. *** indicates p<0.001. **** indicates p<0.0001.

Finally, we assessed the correlation between the mRNA levels of *CD69* and *SBK1* and infiltrating immune cells and IPS. This analysis found that *CD69* mRNA levels positively correlated with immune cell infiltration levels in most tumors (**Figure 6A**, **Supplementary Figure 2A**, **Supplementary Table 4**), and that *SBK1* mRNA levels negatively correlated with tumor infiltration by most immune cells (**Figure 6A**; **Supplementary Figure 2B**; **Supplementary Table 4**). Further analysis of the ability of *CD69* and *SBK1* to predict response to PD-1/PD-L1 blockade cancer immunotherapy using IPS showed that the levels of *CD69* correlated positively with MHC and EC in 31 of 33 tumors, negatively with SC and CP in 32 of 33 tumors, and positively with IPS in 14 of 33 tumors (**Figures 6B**, **7A**, **Supplementary Table 5**). *SBK1* levels correlated negatively with MHC and EC in 18 of 33 tumors, positively with SC and CP in 18 of 33 tumors, and negatively with IPS in 13 of 33 tumors (**Figures 6B**, **7B**, **Supplementary Table 5**). Furthermore, *CD69* levels correlated positively with TMB in 4/33 tumors and negatively in 11/33 tumors (**Supplementary Figure 3A**), while *SBK1* levels correlated positively with TMB in 2/33 tumors and negatively in 4/33 tumors (**Supplementary Figure 3B**).

## Discussion

Although PD-1/PD-L1 blockade exhibits remarkable anticancer efficacy and safety, it is estimated that it benefits less than half of applicable cancer patients (8–10). To better predict response to PD-1/PD-L1 blockade and avoid the waste of medical resources, robust biomarkers have been developed using various strategies, including imaging omics (27, 28) and patient derived biological materials (29–31). Furthermore, genomic based biomarkers, like Tumor Mutational Burden (32), Antigen Processing Machinery score (33), Tumor Immune Dysfunction and Exclusion score (34) and Gene Expression Profiles score (35) are reported as prediction biomarkers. However, the high cost and time-consuming of whole genome sequencing or whole exome sequencing hampered the utility for clinical decision-making (36). Hence, molecular markers that could be applied to improve the prediction accuracy in clinic are still urgently needed. Herein, we sought to develop effective molecular biomarkers for identifying cancer patients who are likely to benefit from PD-1/PD-L1 blockade immunotherapy.

We find that *CD69* and *SBK1* are differentially expressed in cancer patients who respond to PD-1/PD-L1 blockade when compared with non-responders, and that they are potential biomarkers of response to PD-1/PD-L1 blocking cancer immunotherapy. In humans, the *CD69* gene, encodes a disulfide-linked homodimeric protein with two differentially glycosylated subunits (37, 38). Early studies identified CD69 as an early activation marker of various leucocytes, including B cells, T cells, and NK cells (39, 40). However, the roles of CD69 in immune reaction reported by recent studies are controversial. For example, while some studies have implicated the loss of CD69 and autoimmune diseases, others show that CD69 stimulates immune response (41). Here, analysis of CTR-DB data showed that *CD69* is upregulated in lung cancer and melanoma patients who respond to PD-1/PD-L1 blockade, highlighting its potential as a predictor of pan-cancer response to anti-PD-1/PD-L1 immunotherapy.

In contrast, we found that while *SBK1* levels are downregulated in lung cancer patients who respond to anti-PD-1/PD-L1 immunotherapy, it is upregulated in melanoma responders. SBK1 was first identified as a novel serine/threonine kinase in 2001 and named based on its protein structure (42). Human *SBK1* has 4 exons, a 1275 bp open reading frame, and encodes a 424-amino acid protein (43). SBK1 was initially thought to be predominantly expressed in the neurons of the developing brain. However, later studies found that it is widely distributed in various human tissues, including lungs, breasts, and prostate (44). Here, using bioinformatics analysis, we identified *SBK1* as a potential predictor of response to PD-1/PD-L1 blockade cancer immunotherapy. However, this finding requires experimental validation.

To better understand the functions of *CD69* and *SBK1* in the tumor immune microenvironment, we carried out a comprehensive pan-cancer analysis using TCGA data. This analysis showed that *CD69* is aberrantly expressed in most TCGA tumors, and that its expression positively correlated with most immune checkpoints and immune cell infiltration of the tumor microenvironment. A mounting body of evidence indicates that CD69 and its ligand, Myl9, modulate immune responses (45, 46). Mita et al. showed that CD69 could induce the exhaustion of tumor-infiltrating T cells and promote immune escape through a murine 4T1 breast tumor model (47). Furthermore, its blockade might effectively enhance anti-tumor responses (46). However, CD69 expression on memory CD8 T cells is required for cancer cell elimination and the maintenance of cancer-immune equilibrium (48, 49). Thus, CD69 has a double-edged effect in tumor immunity and here, we show that it may effectively predict response to PD-1/PD-L1 blockade.

Our analysis also revealed that *SBK1* upregulation negatively correlates with most tumor-infiltrating immune cells. To our knowledge, only a few studies have examined the role of SBK1 in cancer. Consistent with our findings, SBK1 is reported to be upregulated in OV and to protect OV cells from apoptosis (43). Another study reported that lncRNA ELFN1-AS1 promotes retinoblastoma progression by upregulating SBK1 expression (50). A recent study found that SBK1 plays a key role in lipid metabolism (51). Mechanistically, SBK1 is thought to promote FGF21 expression by phosphorylating Nur77 and suppressing the expression of lipid anabolism genes (51). Thus, we speculate that SBK1 affects the immune landscape by modulating lipid metabolism in the tumor microenvironment, which warrants further investigation.

Finally, we used IPS to assess immunogenicity and response to PD-1/PD-L1 blockade cancer immunotherapy. IPS can predict response to CTLA-4 and PD-1 blockade and has been used in an increasing number of studies (52–54). Here, we show that in various tumors, IPS correlates positively and negatively with *CD69* and *SBK1* expression levels, respectively. These results highlight *CD69* and *SBK1* as potential biomarkers in various cancers. However, the clinical value of this possibility requires further research.

In summary, our study indicates that *CD69* and *SBK1* expression levels can effectively predict cancer response to PD-1/PD-L1 blockade immunotherapy. Such biomarkers can help to guide treatment decisions and avoid the waste of medical resources.

## Data availability statement

The original contributions presented in the study are included in the article/**Supplementary Material**. Further inquiries can be directed to the corresponding authors.

## Ethics statement

Ethical review and approval was not required for the study on human participants in accordance with the local legislation and institutional requirements. Written informed consent from the patients/participants or patients/participants legal guardian/next of kin was not required to participate in this study in accordance with the national legislation and the institutional requirements.

## Author contributions

Study concept: Z-WH, W-BL, W-PW. Data acquisition and processing: Y-HW, L C, W-QC. Data analysis: Z-WH, WS, R-QM. Drafting of manuscript: Z-WH. Critical review and approval of manuscript: all authors.

## Funding

This study was supported by the National Natural Science Foundation of China (NSFC) grants 82020108009, 81870696 (W-PW) and 81972527 (WS), Guangdong Natural Science Foundation of China grant 2018B030312008 (W-PW), Guangdong Research Program of Key Fields in Province 2020B1111300003 (W-PW), the Key-Area Research and Development of Guangdong Province 2020B1111190001 (WPW), and China Postdoctoral Science Foundation 2021M703712 (Z-WH).

## Acknowledgments

We thank The International Postdoctoral Exchange Fellowship Program (Z-WH).

## Conflict of interest

The authors declare that the research was conducted in the absence of any commercial or financial relationships that could be construed as a potential conflict of interest.

The reviewer SW declared a shared affiliation with the authors to the handling editor at time of review.

## Publisher’s note

All claims expressed in this article are solely those of the authors and do not necessarily represent those of their affiliated organizations, or those of the publisher, the editors and the reviewers. Any product that may be evaluated in this article, or claim that may be made by its manufacturer, is not guaranteed or endorsed by the publisher.

## Supplementary material

Supplementary Figure 1 Analysis of the correlation between *CD69* and *SBK1* expression levels and immune checkpoints in all TCGA tumors. **(A)** Correlation between immune checkpoints and the expression levels of *CD69*. **(B)** Correlation between immune checkpoints and the expression levels of *SBK1*. * indicates p<0.05.

Supplementary Figure 2 Analysis of correlation between the levels of *CD69* and *SBK1*, and infiltrating immune cells in all TCGA tumors. **(A)** *CD69* positively correlates with various immune cells in most TCGA tumors. Top left corner: p-value. Lower right corner: correlation coefficient. **(B)** *SBK1* negatively correlates with various immune cells in most TCGA tumors. * indicates (p<0.05).

Supplementary Figure 3 Analysis of correlation between the levels of *CD69* and *SBK1*, and TMB in all TCGA tumors. **(A)** Correlation between TMB and *CD69* levels. **(B)** Correlation between TMB and *SBK1* levels. * indicates p<0.05.

Supplementary Table 1 Detailed list of DEGs in responders versus non- responders.

Supplementary Table 2 The *CD69* and *SBK1*similar expression gene list from GEPIA2.

Supplementary Table 3 Detailed results of the correlation between *CD69* and *SBK1* expression levels and immune checkpoints.

Supplementary Table 4 Detailed results of the correlation between *CD69* and *SBK1* expression levels and infiltrating immune cells.

Supplementary Table 5 Detailed results of the correlation between *CD69* and *SBK1* expression levels and IPS.

## Abbreviations

ACC, Adrenocortical carcinoma; BLCA, Bladder urothelial carcinoma; BRCA, Breast invasive carcinoma; CESC, Cervical squamous cell carcinoma and endocervical adenocarcinoma; CHOL, Cholangiocarcinoma; COAD, Colon adenocarcinoma; DLBC, Lymphoid neoplasm diffuse large B-cell lymphoma; ESCA, Esophageal carcinoma; GBM, Glioblastoma multiforme; HNSC, Head and neck squamous cell carcinoma; KICH, Kidney chromophobe; KIRC, Kidney renal clear cell carcinoma; KIRP, Kidney renal papillary cell carcinoma; LAML, Acute myeloid leukemia; LGG, Brain lower grade glioma; LIHC, Liver hepatocellular carcinoma; LUAD, Lung adenocarcinoma; LUSC, Lung squamous cell carcinoma; MESO, Mesothelioma; OV, Ovarian serous cystadenocarcinoma; PAAD, Pancreatic adenocarcinoma; PCPG, Pheochromocytoma and paraganglioma; PRAD, Prostate adenocarcinoma; READ, Rectum adenocarcinoma; SARC, Sarcoma; SKCM, Skin cutaneous melanoma; STAD, Stomach adenocarcinoma; TGCT, Testicular germ cell tumors; THCA, Thyroid carcinoma; THYM, Thymoma; UCEC, Uterine corpus endometrial carcinoma; UCS, Uterine carcinosarcoma; UVM, Uveal melanoma.
