# Supplementary figures and images for: CD69 and SBK1 as potential predictors of responses to PD-1/PD-L1 blockade cancer immunotherapy in lung cancer and melanoma
Source: Front Immunol. 2022 Aug 15;13:952059. doi: 10.3389/fimmu.2022.952059 (PMC9421049; doi:10.3389/fimmu.2022.952059)

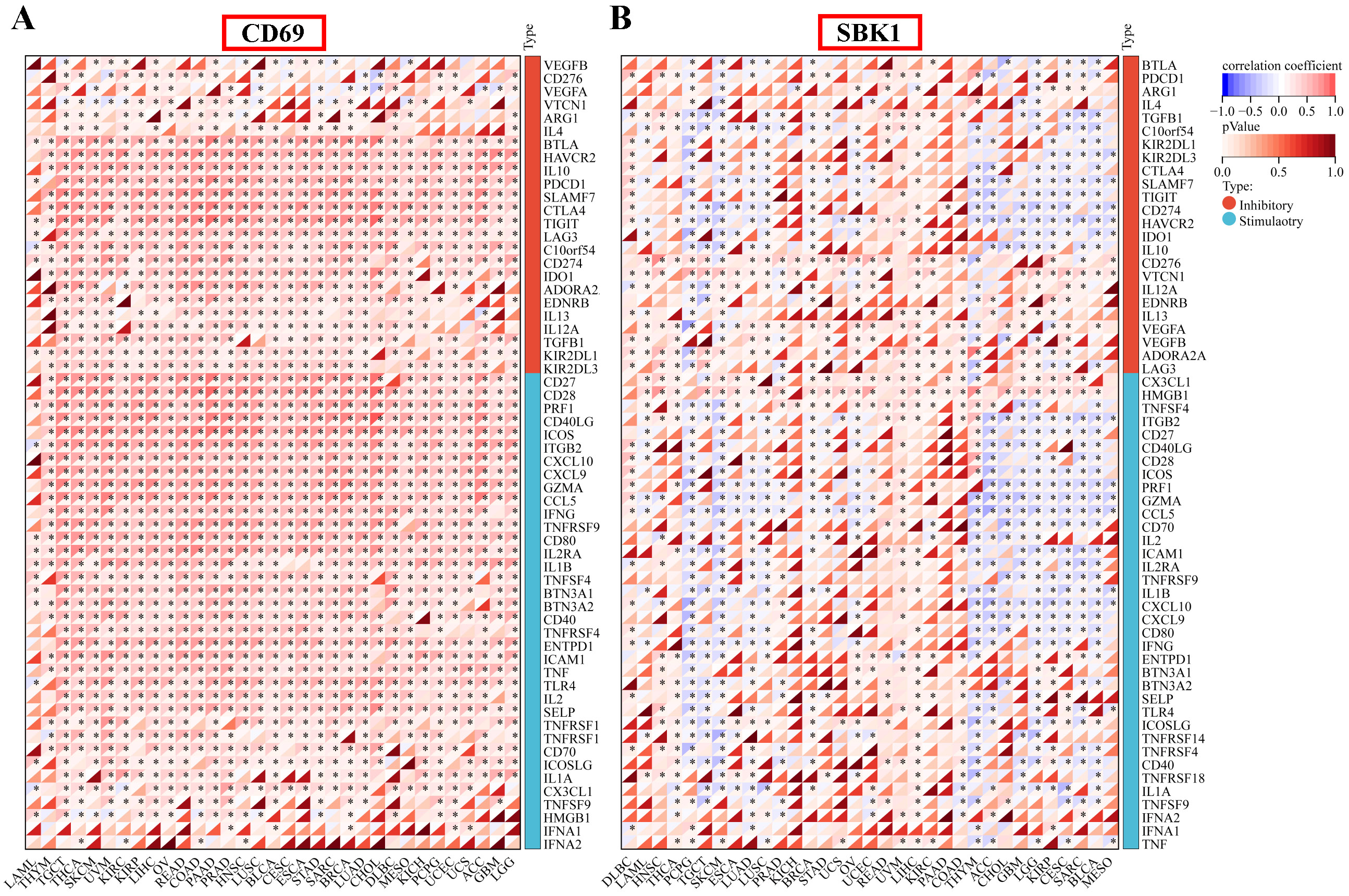

Supplement: Supplementary Figure 1 — Analysis of the correlation between CD69 and SBK1 expression levels and immune checkpoints in all TCGA tumors. (A) Correlation between immune checkpoints and the expression levels of CD69. (B) Correlation between immune checkpoints and the expression levels of SBK1. * indicates p<0.05. [file Image_1.jpeg]

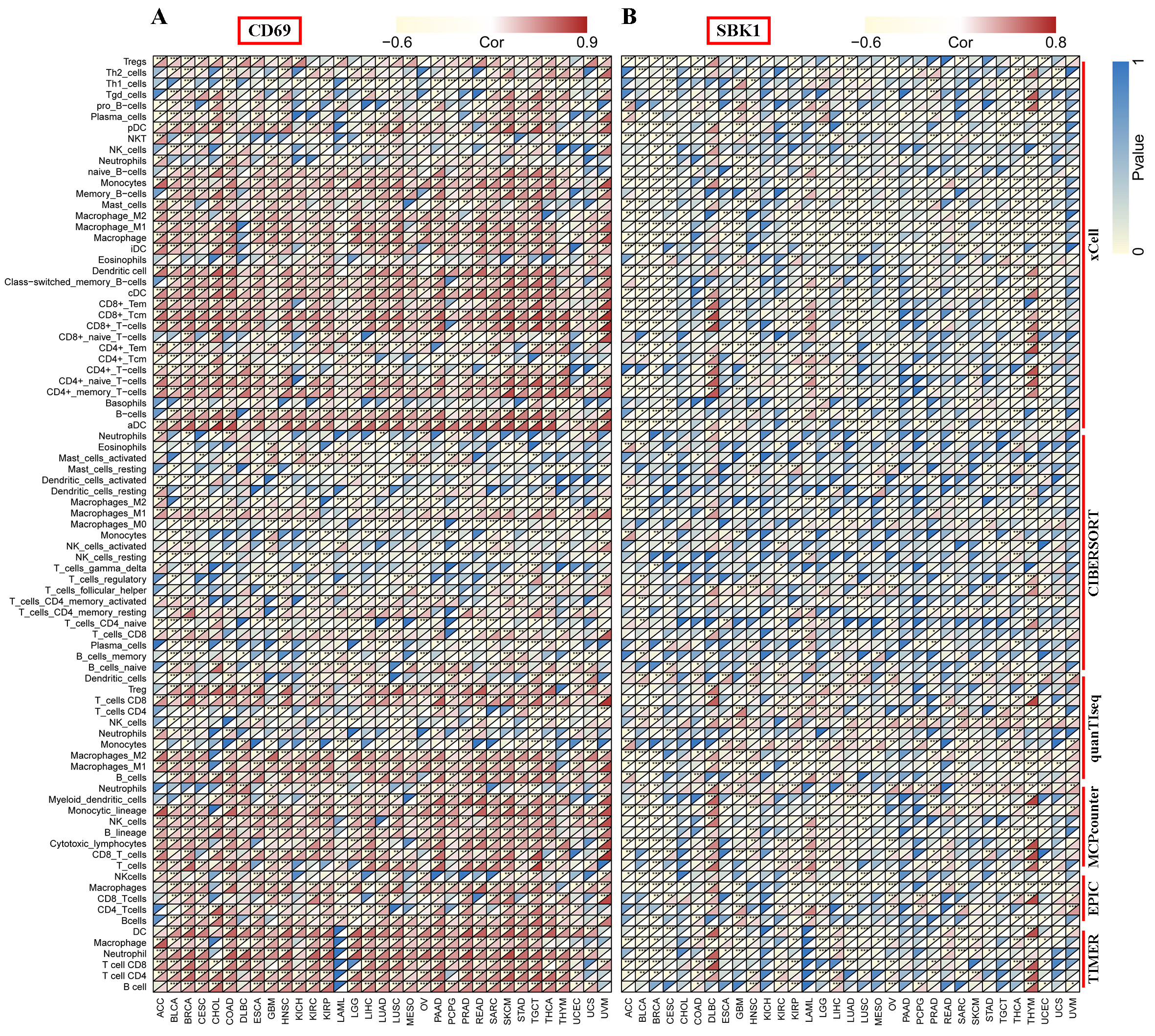

Supplement: Supplementary Figure 2 — Analysis of correlation between the levels of CD69 and SBK1, and infiltrating immune cells in all TCGA tumors. (A) CD69 positively correlates with various immune cells in most TCGA tumors. Top left corner: p-value. Lower right corner: correlation coefficient. (B) SBK1 negatively correlates with various immune cells in most TCGA tumors. * indicates (p<0.05). [file Image_2.jpeg]

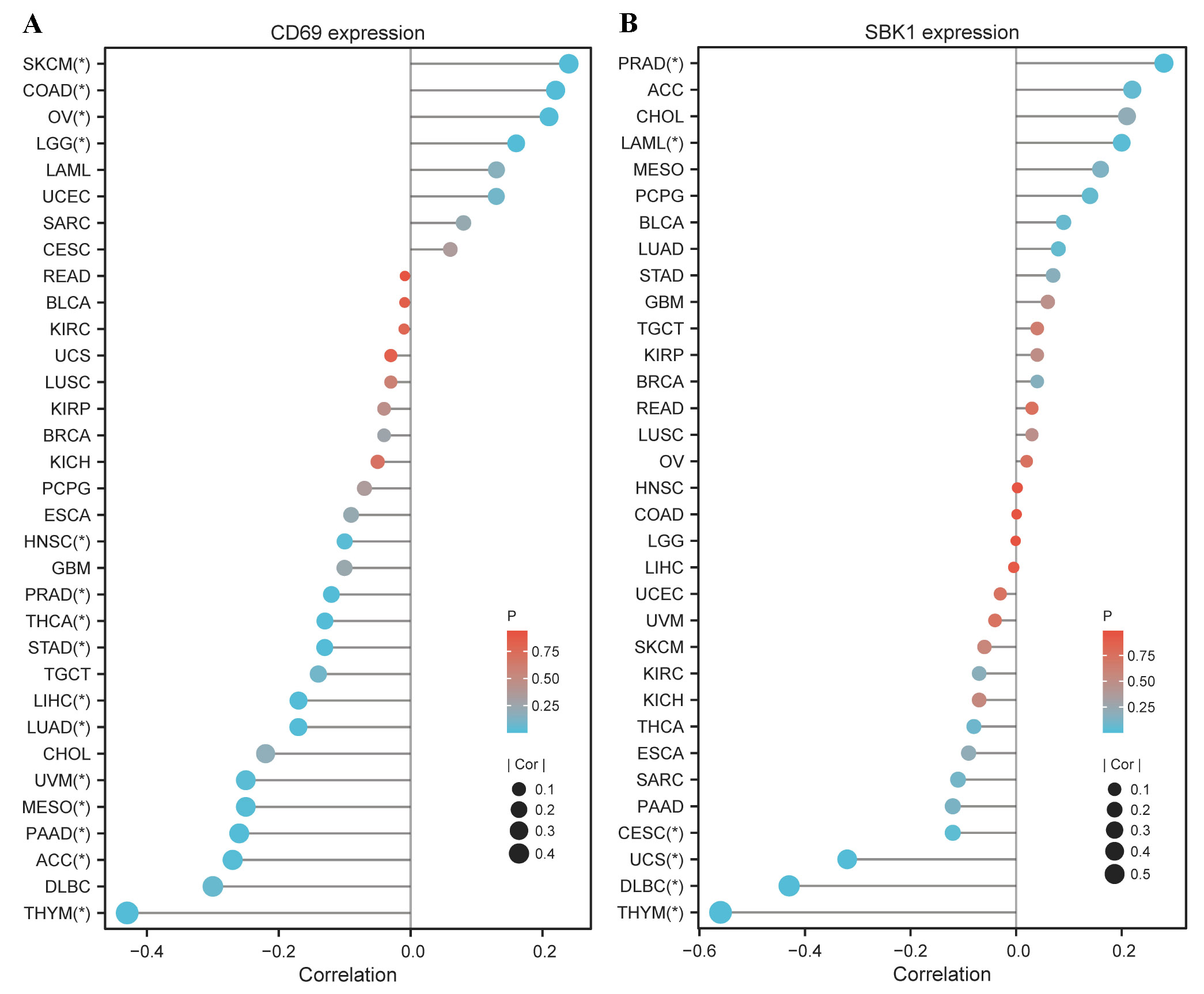

Supplement: Supplementary Figure 3 — Analysis of correlation between the levels of CD69 and SBK1, and TMB in all TCGA tumors. (A) Correlation between TMB and CD69 levels. (B) Correlation between TMB and SBK1 levels. * indicates p<0.05. [file Image_3.jpeg]
